# Rapid and Highly Informative Diagnostic Assay for H5N1 Influenza Viruses

**DOI:** 10.1371/journal.pone.0000095

**Published:** 2006-12-20

**Authors:** Nader Pourmand, Lisa Diamond, Rebecca Garten, Julianna P. Erickson, Jochen Kumm, Ruben O. Donis, Ronald W. Davis

**Affiliations:** 1 Stanford Genome Technology Center, Stanford University, Palo Alto, California, United States of America; 2 Molecular Virology and Vaccines Branch, Influenza Division, Centers for Disease Control and Prevention, Atlanta, Georgia, United States of America; University of Cambridge, United Kingdom

## Abstract

A highly discriminative and information-rich diagnostic assay for H5N1 avian influenza would meet immediate patient care needs and provide valuable information for public health interventions, e.g., tracking of new and more dangerous variants by geographic area as well as avian-to-human or human-to-human transmission. In the present study, we have designed a rapid assay based on multilocus nucleic acid sequencing that focuses on the biologically significant regions of the H5N1 hemagglutinin gene. This allows the prediction of viral strain, clade, receptor binding properties, low- or high-pathogenicity cleavage site and glycosylation status. H5 HA genes were selected from nine known high-pathogenicity avian influenza subtype H5N1 viruses, based on their diversity in biologically significant regions of hemagglutinin and/or their ability to cause infection in humans. We devised a consensus pre-programmed pyrosequencing strategy, which may be used as a faster, more accurate alternative to *de novo* sequencing. The available data suggest that the assay described here is a reliable, rapid, information-rich and cost-effective approach for definitive diagnosis of H5N1 avian influenza. Knowledge of the predicted functional sequences of the HA will enhance H5N1 avian influenza surveillance efforts.

## Introduction

The worldwide spread of high-pathogenicity H5N1 avian influenza A virus in poultry and wild birds has resulted in many human infections, with high fatality rates. Although sustained human-to-human transmission has not yet occurred, concern about a potential pandemic continues to mount. The current HA lineage of H5N1 avian influenza was first found among domestic poultry populations in 1996 in southern China [1]. A similar H5N1 influenza virus spread directly from poultry to humans in Hong Kong in 1997, causing death in 6 out of 18 persons diagnosed with infection with this virus [2]. While the massive culling of poultry in 1997 temporarily eradicated the virus in Hong Kong, the virus has continued to spread across Asia, causing human deaths in Thailand, Vietnam, Indonesia, China and elsewhere [2], [3]. The rapid spread of H5N1 in birds from Asia into Europe and Africa in recent months has intensified efforts to control the virus and avert a pandemic. To address the recognized need for rapid, low-cost diagnosis, tracking critically important genetic changes in the virus among animal and human host populations, and identifying specific viral clades [4], we have developed high-throughput methods for monitoring viral mutations that may control virulence and transmissibility in humans [5]. Accurate and rapid detection and tracking of H5N1 will be critical to prevent or control a potential pandemic.

Diagnosis of influenza type A infections in clinical microbiology laboratories has traditionally been performed using cell culture and/or direct fluorescent antibody assays [5]–[7]. These methods are time-consuming and require biosafety level 3 enhanced biocontainment facilities and equipment to protect laboratory personnel from exposure to H5N1 cultured in the laboratory. Because these facilities not widely available, culture-based assays are increasingly being replaced in clinical settings by the various polymerase chain reaction (PCR) methods [8]–[11].

**Figure 1:**
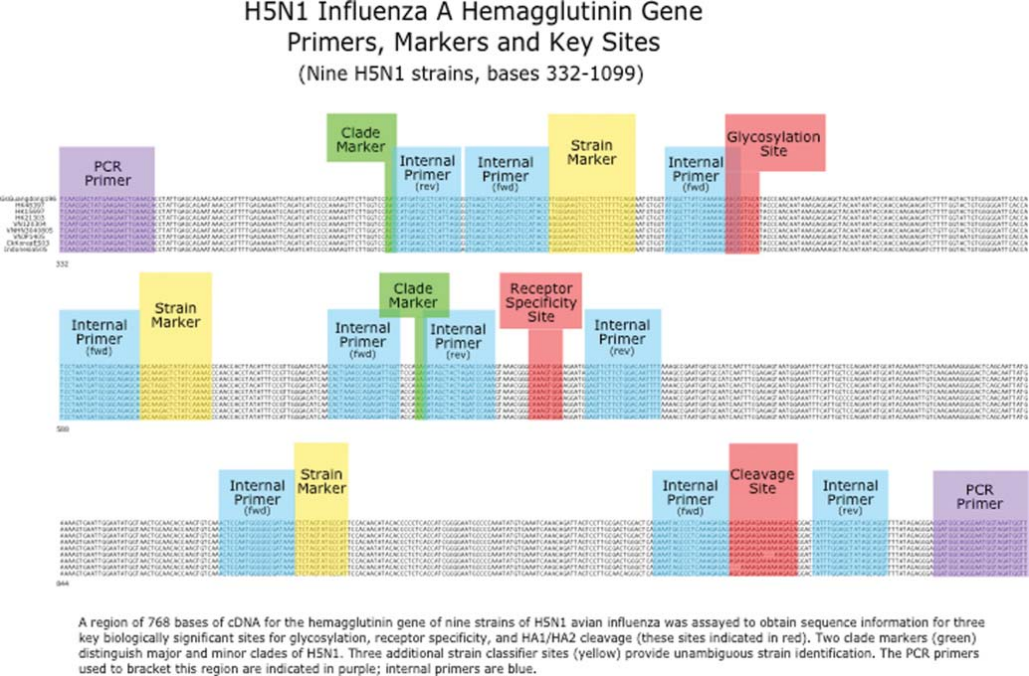
Primers, markers and key sites on H5N1 influenza A hemagglutinin gene. A region of 768 bases of cDNA of the hemagglutinin gene of nine strains of H5N1 was assayed to obtain sequence information for three key biologically significant sites (glycosylation, receptor specificity, and HA1/HA2 cleavage; (sites indicated in red)). Two markers (green) distinguish clades of H5N1. Three additional polymorphic sites (yellow) provide unambiguous strain identification. The PCR primers used to bracket this region are indicated in purple; internal primers are blue.

PCR is more sensitive than traditional tests and detection does not require viable virus or morphologically intact infected cells in the sample. A PCR-based molecular diagnostic test is currently the most widely used by public health laboratories to diagnose the presence of H5N1 in clinical specimens [12]. We hypothesized that coupling a PCR assay to a rapid sequencing method would further increase the value of molecular techniques for virus identification and characterization, especially if implemented into automated robotic platforms in the near future. Nucleic acid sequencing is considered the most reliable and highest-resolution method for virus identification, but is typically too slow and costly to use as a primary assay. Samples can be prepared sequentially for PCR diagnosis of H5N1 influenza virus, and pyrosequencing, yielding results in approximately 90 minutes, with immediate availability of the viral sequence data. The speed, sensitivity, precision, low cost, and high throughput of this method give it substantial advantages in H5N1 influenza characterization. We have designed an assay that focuses on three biologically significant regions of the H5N1 hemagglutinin gene (Figure 1), including sites informative of viral ancestry.

Influenza type A viruses have an eight-segment negative-sense RNA genome complexed with nucleoprotein and polymerase surrounded by the matrix protein and a lipid envelope that contains two integral membrane glycoproteins, hemagglutinin (HA) and neuraminidase (NA), protruding from the virion surface. The HA binds to sialic acid-terminated glycan receptors on the host cell surface, triggering virion uptake by endocytosis. Human respiratory tract cells have predominantly alpha 2-6 linked sialic acid receptors, whereas duck intestine has predominantly alpha 2-3 linked sialic acid receptors. Host-adapted human and avian influenza viruses selectively bind to homologous variant sialoside structures [13]–[15]. The HA receptor binding site is located at the convergence of one helix, two loops and three single residues near the top of the molecule; amino acid residues in the 184-186 (188-190 H3) helix as well as the 130–134 (134-138 H3) and 217–224 (221-228 H3) loops make up the rims, whereas residues 91 (98 H3), 149 (153 H3) and 179 (183 H3) form the floor of the cavity (H5 numbering; references: [16], [17]). In the H3 subtype of HA, amino acids 222 and 224 (226 and 228 H3) are major determinants of human or avian host sialic acid specificity of the virus; alpha 2-6 linkages in human viruses and alpha 2-3 linkages in avian viruses [18]. Similar changes have been documented for the H1 HA that caused the 1918 so-called Spanish influenza pandemic [19], [20]. Mutation at amino acid 223 from serine-to-asparagine (227 in H3) in a 2003 human H5N1 isolate (A/Hong Kong/213/03) maintained avian-like binding specificity of alpha 2-3 linked sialic acids but also had increased binding to alpha 2-6 sialic acid linkages [17].

The HA is also responsible for cell entry by mediating fusion of the endosomal and viral membranes. The HA requires proteolytic cleavage to become functional in membrane fusion. Cleavage, mediated by host proteases, results in two disulfide-linked subunits, HA1 and HA2. The HA1 region of the HA gene encompasses three sites of known biological significance:

- A glycosylation sequon at the top of the receptor binding site at amino acids 154-156 (of mature H5 HA1) (158-160 H3) has been linked to viral adaptation from waterfowl to domesticated poultry. The majority of H5N1 viruses isolated from humans since 2004 have this motif [4], [21].
- The receptor binding site. Changes in the rims or floor of the receptor-binding site thought to favor binding of human forms of cell surface sialosides are considered critical for sustained transmission in the human population.
- Finally, the number of basic amino acids (lysine or arginine) in the cleavage site between HA1 and HA2 determines whether the virus is highly pathogenic for birds [22], [23]. All Eurasian H5N1 viruses currently circulating are highly pathogenic and contain either five or six consecutive basic amino acids at this site [4].

Recent phylogenetic analysis of H5N1 evolution indicates that distinct major and minor clades have emerged among HA gene lineages; the two most important ones of these from a public health perspective are termed clades 1 and 2. These two clades are identifiable with two amino acids, 124 and 212 (129 and 216 in H3) [4]. These clade distinctions may be antigenically significant; therefore clade identification may aid selection of appropriate vaccines. Rapid H5N1 identification will assist in pinpointing the source of particularly virulent outbreaks, and in targeting limited supplies of vaccines and anti-virals to key regions.

Here, we report the results of a proof-of-principle experiment based on the application of pyrosequencing technology targeting the hemagglutinin gene of H5N1 influenza. The assay uses RT-PCR to amplify a known H5N1-specific region of 768 nucleotides. Subsequent pyrosequencing [24] of strains that are H5N1-positive with ten specific sequencing primers is used to determine HA clade and strain, receptor binding preference, low- or high-pathogenicity cleavage site, and glycosylation status. The assay presented here is considerably more informative than traditional techniques such as real-time PCR, as it not only identifies the H5N1 lineage but also predicts receptor binding properties that could herald the development of human-human transmissibility. This assay would be most advantageous as part of a screening method during high-volumes of H5N1 activity. Also, pyrosequencing has some advantages over Sanger sequencing-based methods, specifically the availability of sequence data directly following the sequencing primer, and the accessibility of the results in real time. Moreover, this assay is specific, rapid and cost-effective.

## Results

### HA Amplification by PCR

**Figure 2:**
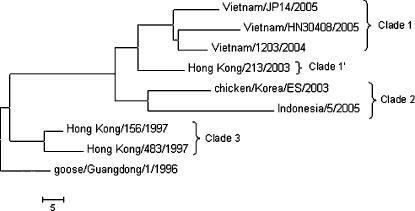
Phylogenetic relationships of H5N1 hemagglutinin (HA) genes from highly pathogenic H5N1 avian influenza viruses used in this study. Phylogenetic trees were inferred from nucleotide sequences by the neighbor joining method in the MEGA program. Horizontal distances are proportional to the number of nucleotide changes between the viruses. HA clade determinations are shown on the right.

**Figure 3:**
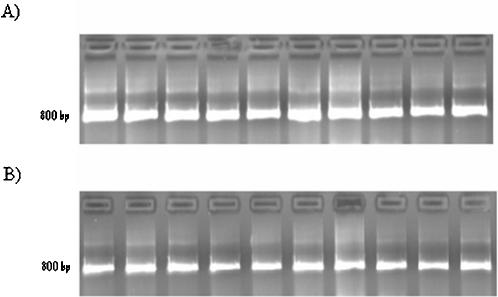
Amplicon DNA yield from PCR with different combinations of biotinylated primers. All PCR products were Sanger-sequenced to confirm the identity of the sample. A) The nine H5N1 samples amplified with a biotinylated forward primer (B-F-H5N1-1) and nonbiotinylated reverse primer (R-H5N1-2). From left to right, the samples are A/goose/Guangdong/1/96 (AF144305), A/Hong Kong/156/97 (AF036356), A/Hong Kong/483/97 (AF046097), A/Hong Kong/213/2003 (AY575869), A/chicken/Korea/ES/03 (AY676035), A/Vietnam/1203/2004 vaccine candidate with deletion of polybasic cleavage site (AY651334), A/Vietnam/JP14/2005 (ISDN117778), A/Vietnam/HN30408/2005 (ISDN119678), A/Indonesia/5/05 (ISDN125873). B) The H5N1 PCR products amplified with a biotinylated reverse primer (B-R-H5N1-3) and a nonbiotinylated forward primer (F-H5N1-4). Samples are in the order stated for part (A).

The first step in the analysis of a clinical specimen or a viral isolate in our assay is the generation of a DNA copy of the viral RNA, which is accomplished by reverse transcription coupled to PCR (RT-PCR). This was performed using two different biotinylated combinations of PCR primers specific to the H5N1 regions of interest in order to achieve optimal sequencing flexibility of H5N1 isolates, as listed in Figure 2. As shown in Figure 3, either pair of primers provided reliable amplification of H5N1, and neither generated products when used in PCRs with negative controls (total genomic DNA from unrelated human cell lines) (confirmed by Sanger sequencing).

**Figure 4:**
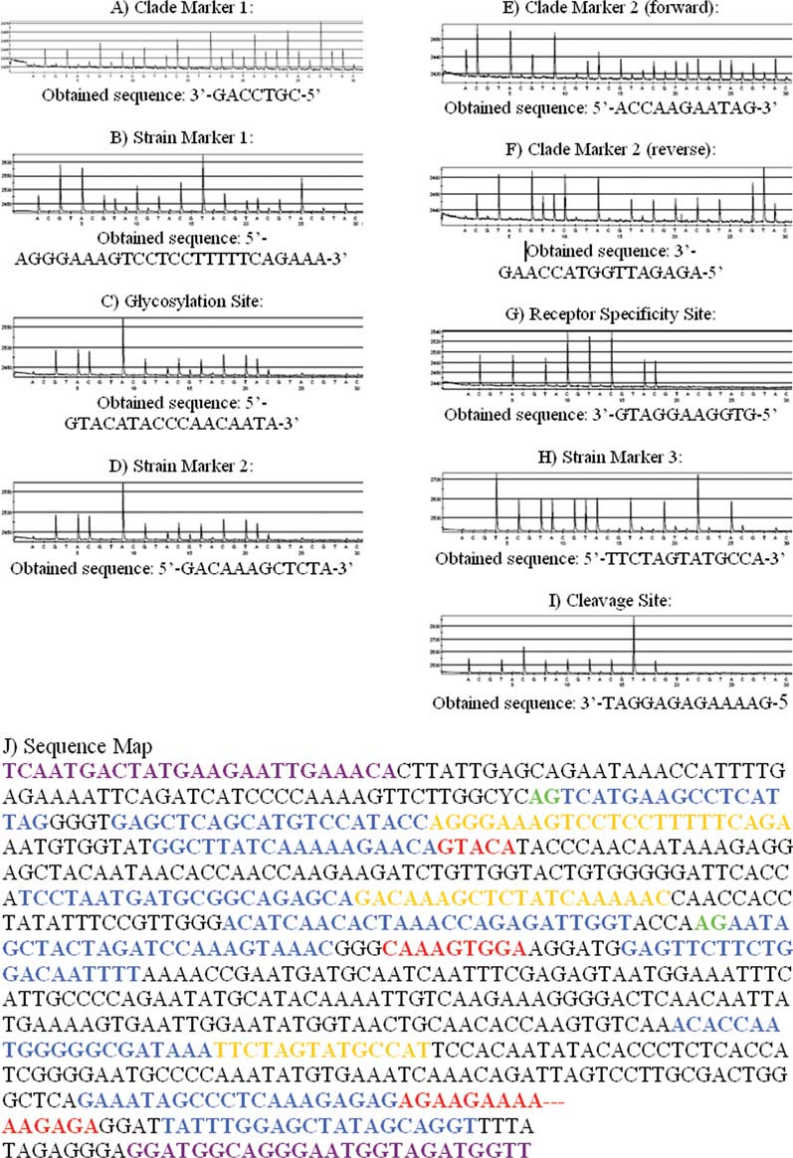
Panels A–I: Pyrograms obtained by pyrosequencing of sample A/Vietnam/HN30408/2005 with the HS 96 system. Peaks above a given nucleotide sample indicate nucleotide incorporation (with height proportional to number of consecutive incorporation events). Initial pyrosequencing was performed with systematic nucleotide dispensation for *de novo* sequencing (pre-programmed dispensation runs can also be performed). Panel J: Map of the HA sequence of A/Vietnam/HN30408/2005 obtained by Sanger sequencing; pyrosequencing data is overlaid and color-coded to show correlation between the sequence data obtained by the two methods. Reverse-primed pyrosequencing results should be read as reverse complements. For clarity, primers and biologically significant sequence segments are colored to correspond to the sequence map presented in Figure 1.

All of the H5N1 PCR products were sequenced at least twice both by Sanger dideoxy sequencing and pyrosequencing; PCR-positive amplicons generated correct sequence results independent of sequencing method or fragment size. Furthermore, no loops or primer-dimers were observed when primers were pyrosequenced in the absence of template. Representative and typical pyrograms of sample A/Vietnam/HN30408/ 2005, obtained by pyrosequencing, are shown in Figure 4; pyrograms for the remaining eight samples are available as supplementary information. The pyrosequencing run covering the required 14 bases took approximately 15 minutes.

### HA Pyrosequencing

The results of our pyrosequencing assay clearly distinguished the nine different strains of H5N1 avian influenza, based on eight sites, as shown in Table 1. Furthermore, this approach provided accurate sequencing of regions of known biological significance (for supplementary data, see http://bioel.stanford.edu/avianflu/index.html). The results of this assay can be seen in Table 2, which shows the amino acid sequences characteristic of each strain tested.

**Table 1:**
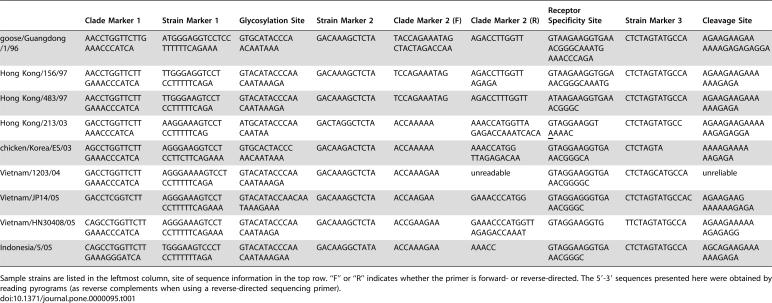
Sequences of H5N1 clade markers and active sites, as determined by pyrosequencing.

	Clade Marker 1	Strain Marker 1	Glycosylation Site	Strain Marker 2	Clade Marker 2 (F)	Clade Marker 2 (R)	Receptor Specificity Site	Strain Marker 3	Cleavage Site
goose/Guangdong/1/96	AACCTGGTTCTTGAAACCCATCA	ATGGGAGGTCCTCCTTTTTTCAGAAA	GTGCATACCCAACAATAAA	GACAAAGCTCTA	TACCAGAAATAGCTACTAGACCAA	AGACCTTGGTT	GTAAGAAGGTGAAACGGGCAAATGAAACCCAGA	CTCTAGTATGCCA	AGAAGAAGAAAAAAGAGAGAGGA
Hong Kong/156/97	AACCTGGTTCTTGAAACCCATCA	TTGGGAGGTCCTCCTTTTTCAGA	GTACATACCCAACAATAAAGA	GACAAAGCTCTA	TCCAGAAATAG	AGACCTTGGTTAGAGA	GTAAGAAGGTGGAAACGGGCAAATG	CTCTAGTATGCCA	AGAAGAAGAAAAAAGAGA
Hong Kong/483/97	AACCTGGTTCTTGAAACCCATCA	TTGGGAAGTCCTCCTTTTTCAGA	GTACATACCCAACAATAAAGA	GACAAAGCTCTA	TCCAGAAATAG	AGACCTTTGGTT	ATAAGAAGGTGAAACGGGC	CTCTAGTATGCCA	AGAAGAAGAAAAAAGAGA
Hong Kong/213/03	GACCTGGTTCTTAAACCCATCA	AAGGAAAGTCCTCCTTTTTCAG	ATGCATACCCAACAATAA	GACTAGGCTCTA	ACCAAAAA	AAACCATGGTTAGAGACCAAATCACA	GTAGGAAGGTAAAAC	CTCTAGTATGCC	AGAAGAAGAAAAAAGAGAGGA
chicken/Korea/ES/03	AGCCTGGTTCTTGAAACCCATCA	AGGGAAGGTCCTCCTTCTTCAGAAA	GTGCACTACCCAACAATAAA	GACAAGACTCTA	ACCAAAAA	AAACCATGGTTAGAGACAA	GTAGGAAGGTGAAACGGGCA	CTCTAGTA	AAAAGAAAAAAGAGA
Vietnam/1203/04	GACCTGGTTCTTGAAACCCATCA	AGGGAAAAGTCCTCCTTTTTCAGA	GTACATACCCAACAATAAAGA	GACAAAGCTCTA	ACCAAAGAA	unreadable	GTAGGAAGGTGAAACGGGGC	CTCTAGCATGCCA	unreliable
Vietnam/JP14/05	GACCTCGGTCTT	AGGGAAAGTCCTCCTTTTTCAGAAA	GTACATACCAACAATAAAGAAA	GACAAAGCTCTA	ACCAAGAA	GAAACCCATGG	GTAGGAGGGTGAAACGGGC	CTCTAGTATGCCAC	AGAAGAAGAAAAAAGAGA
Vietnam/HN30408/05	CAGCCTGGTTCTTGAAACCCATCA	AGGGAAAGTCCTCCTTTTTCAGAAA	GTACATACCCAACAATAAGA	GACAAAGCTCTA	ACCGAAGAA	GAAACCCATGGTTAGAGACCAAAT	GTAGGAAGGTG	TTCTAGTATGCCA	AGAAGAAAAAAGAGAGG
Indonesia/5/05	CAGCCTGGTTCTTGAAAGGGATCA	TGGGAAGTCCCTCCTTTTTTAGA	GTACATACCCAACAATAAAGAA	GACAAGGCTATA	ACCAAAGAA	AAACC	GTAGGAAGGTGAAACGGGC	CTCTAGTATGCCA	AGCAGAAGAAAAAAGAGA

Sample strains are listed in the leftmost column, site of sequence information in the top row. “F” or “R” indicates whether the primer is forward- or reverse-directed. The 5′-3′ sequences presented here were obtained by reading pyrograms (as reverse complements when using a reverse-directed sequencing primer).

**Table 2:**
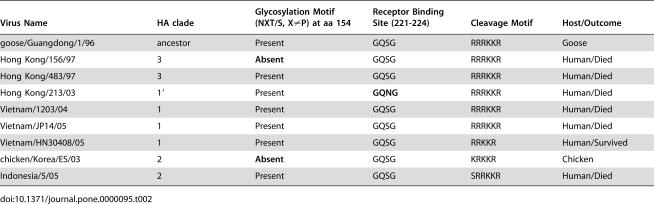
Characterization of the hemagglutinin from the H5N1 influenza viruses used in this study.

Virus Name	HA clade	Glycosylation Motif (NXT/S, X≠P) at aa 154	Receptor Binding Site (221-224)	Cleavage Motif	Host/Outcome
goose/Guangdong/1/96	ancestor	Present	GQSG	RRRKKR	Goose
Hong Kong/156/97	3	**Absent**	GQSG	RRRKKR	Human/Died
Hong Kong/483/97	3	Present	GQSG	RRRKKR	Human/Died
Hong Kong/213/03	1′	Present	**GQNG**	RRRKKR	Human/Died
Vietnam/1203/04	1	Present	GQSG	RRRKKR	Human/Died
Vietnam/JP14/05	1	Present	GQSG	RRRKKR	Human/Died
Vietnam/HN30408/05	1	Present	GQSG	RRKKR	Human/Survived
chicken/Korea/ES/03	2	**Absent**	GQSG	KRKKR	Chicken
Indonesia/5/05	2	Present	GQSG	SRRKKR	Human/Died

In order to characterize the strains based on the HA sequence, pyrograms obtained via pyrosequencing were visually inspected to verify the sequences of functionally relevant sites and the presence of polymorphisms that could serve as lineage markers. These sequences were then compared to known H5N1 sequences for clade and strain identification. Certain positions provided essential information for the identification of signatures important for public health, such as changes in the receptor binding site which could signal an increase in the ability of the virus to transmit from human- to-human. The prototype receptor binding site for avian isolates in amino acids 221-224 of the HA1 is GQSG. One human isolate, Hong Kong/213/03, has a mutation at amino acid 223, S->N, which switches the affinity of the hemagglutinin from alpha 2-3 linked sialic acid to the alpha 2-6 linked sialic acid preferred by human influenza viruses. This mutation was detected by a single nucleotide change, G->A, in the sequence of the receptor binding active site. Rapid characterization of the receptor binding active site and other active sites of the hemagglutinin is critical in identifying viruses with increased pandemic potential.

## Discussion

Our initial results indicate that targeted pyrosequencing approach can clearly distinguish among different strains of H5N1, and can accurately sequence regions of known biological significance such as receptor binding (supplementary data: http://bioel.stanford.edu/avianflu/index.html). The HA sequence results obtained by pyrosequencing were 100% identical to those obtained by the Sanger method (two replicates were performed to cross-verify results). Three pyrosequencing replicates, with nine samples at ten sites, were performed: two with a *de novo* dispensation and one with a pre-programmed dispensation order. Of the three pyrosequencing replicates performed, all successful runs verified one another. Because Sanger sequencing relies on molecular separation of the polymerase-mediated extension of the primer, the first ∼20–50 bases 3′ of the primer are not discernible. In contrast, pyrosequencing provides unambiguous sequence from the first nucleotide 3′ to the primer, increasing the sequence information yield per assay. This feature of pyrosequencing was exploited by designing H5N1-specific primers adjacent to critical polymorphic sites such that the initial base-callings are usually sufficient to determine the lineage of a given HA gene.

A simpler detection assay giving a positive or negative identification of H5N1 will be helpful, but a more information-rich method is necessary to provide additional information to guide patient care or public health measures, especially as new strains emerge. Indeed, some laboratories are already using pyrosequencing to identify drug-resistant viruses [25]. As H5N1 influenza becomes more prevalent, it will be impractical to perform full sequencing of all isolates in time for analysis of rapidly changing epidemiological trends. Our assay could be a valuable complement to full sequencing at public health laboratories. We have developed a rapid and inexpensive assay based on DNA sequencing for early detection of virus present in host cells. This assay permits rapid, simplified and accurate identification of avian H5N1 influenza A, and consists of simple procedures maintaining high sensitivity and specificity. As new sites of functional relevance are identified, new site-specific primers can be added to broaden the utility of the assay. Expanded knowledge of H5N1 sequence and evolution is likely to contribute to more effective diagnostic methods and treatments that require less investment of time and money.

An additional advantage of pyrosequencing over the Sanger method is its application in genotyping single and multiple HPV infections [26]. This technology is based on the use of multiple sequencing primers in the same pyrosequencing reaction. Pools of primers are chosen based on their position on the target, the sequence information they yield, and their cross-reactivity with other primers in the multiplex assay. Clinically relevant samples containing multiple infections can then be identified and characterized.

**Figure 5:**
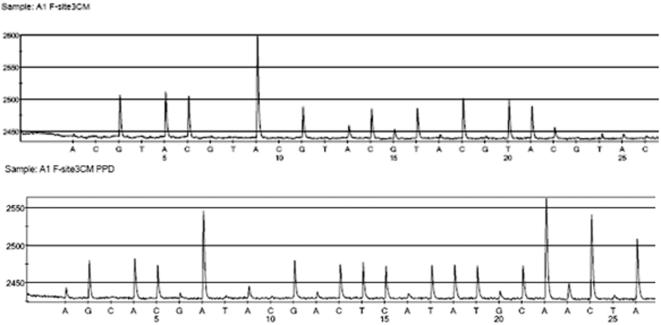
A representative pyrogram illustration. Comparison of pyrosequencing results obtained using the *de novo* (top) and consensus pre-programmed (bottom) sequencing methods for site 3 (a clade marker) in sample A1 (goose/Guangdong/1/96). Both methods give the sequence **GACAAAGCTCTATCAAAAC** for our laboratory stock. In contrast the GenBank sequence (accession # AF144305) reads **GACAAAGCTATATCAAAAC. Database information was found to be based off wild-type sequence information, while the sample we analyzed was obtained via reverse genetics.** A longer read length is obtained via the consensus pre-programmed dispensation method (22 versus 12 nucleotides).

These early robust results have validated our approach and selection of specific primers. We have moved toward a further refinement of the assay based on these results. After an initial *de novo* pyrosequencing run was performed on the H5N1 samples, sequence data for each strain were compiled for each nucleotide site. This information was integrated to produce consensus pre-programmed dispensation orders of nucleotides. This type of pyrosequencing would allow us to obtain sequence results of similarly high quality in 10 to 15 minutes (Figure 5). With this approach, all of the H5N1 subtypes included in this study could be positively identified. New variants would not be fully characterized, but would be heralded by truncated sequences.

In summary, we have developed a reliable, rapid, cost-effective, and information-rich diagnostic assay for H5N1 influenza. This sequence-based method could be extended to include amplicons from other genes of interest; for example, drug targets such as NA and the M2 ion channel [27], [28], or the postulated virulence motifs on NS1 or PB2 [29], [30]. Pyrosequencing is user-friendly, and permits significantly more efficient and rapid genotyping than traditional techniques. This method detects a wide range of influenza A H5N1 subtypes based on sequence information. The assay will allow further development of technology to directly detect H5N1 or specific strains of influenza A in clinical specimens without extensive sample preparation.

## Materials and Methods

### Avian Influenza A Subtypes and H5N1-Specific Primer Design

Sequences of 362 avian influenza A virus species were acquired from GenBank at the National Center for Biotechnology Information and Influenza Sequence Database at Los Alamos National Laboratory and aligned using Clustal X version 1.83 [31]. A specific sequencing primer was designed for H5N1, with no sequence similarity to the other influenza virus HA subtypes based on the database searches and alignments (Figure 1).

### H5N1 Avian Influenza Virus Isolates

The viruses that formed the test set for this study were chosen on the basis of their diversity in biologically significant regions of hemagglutinin and/or their ability to cause infection in humans. Virus strain names are shown in Figure 2 and accession numbers are as follows: A/goose/Guangdong/1/96 (AF144305), A/Hong Kong/156/97 (AF036356), A/Hong Kong/483/97 (AF046097), A/Hong Kong/213/2003 (AY575869), A/chicken/Korea/ES/03 (AY676035), A/Vietnam/1203/2004 vaccine candidate with deletion of polybasic cleavage site (AY651334), A/Vietnam/JP14/2005 (ISDN117778), A/Vietnam/HN30408/2005 (ISDN119678), A/Indonesia/5/05 (ISDN125873).

### RNA Extraction, RT-PCR and PCR Amplification

Viruses were isolated and propagated by inoculation into the allantoic sac of 10-day old chicken embryos, as described previously [32]. Viral infectivity was determined by endpoint dilution, injection into chicken embryos and hemagglutination assay. All work involving infectious H5N1 influenza was performed in government-approved biosafety level 3-enhanced containment facilities as required by the U.S. Department of Agriculture and the Select Agent Program (see guidance at http://www.cdc.gov/flu/h2n2bsl3.htm).Viral RNA was extracted using the QIAmp vRNA Kit (Qiagen, Valencia CA). Extractions were performed according to manufacturer's instructions. QIAGEN Onestep RT-PCR kit (Qiagen, Valencia CA) was used to perform RT-PCR from 30 ng of RNA (approximate concentration of 10 ng/µl by spectrophotometer) in a 50 µl reaction volume. The RT-PCR amplification primers, which are biotinylated F-H5N1-1/3 (5′-TCAAYGACTATGAAGAAYTGAAACA-3′), and R-H5N1-2/4 (5′- AACCATCTACCATTCCCTGCCATCC-3) were synthesized by IDT (Coralville, IA, USA). RT-PCR was performed with a DNA Engine (PTC-200) Peltier Thermal Cycler (BIO-RAD, Hercules, Calif.) as follows: 50°C for 10 minutes, 95°C for 15 minutes, 35 cycles of 95°C, 55°C, and 72°C for 1 minute each, and finally a 10 minute final extension at 72°C. To confirm proper amplification, PCR products were electrophoresed in a 1% agarose gel and visualized by ethidium bromide staining under UV illumination.

### Sanger dideoxy DNA Sequencing of PCR Products

The amplified DNA from all isolates was cycle sequenced in both directions using the BigDye Terminators Reaction Kit v. 3.1 (Applied Biosystems, Foster City, CA) on an ABI automated DNA sequencer (3730 XL DNA Analyzer).

### Hemagglutinin Sequencing Primers

An entropy-based analysis of genetic variation among H5N1 strains (Figure 1) was used to design PCR primers [33]. The approach focused on amplicons comprising regions for receptor binding sites, cleavage site and glycosylation sites, along with markers to identify clade and individual strain (Figure 1). Highly conserved sites were selected as internal sequencing primers to sequence each region of interest. The selected region is bracketed by sequences unique to and highly conserved in the H5N1 subtype. Based on sequence alignments of H5N1 and sequencing results (data not shown) from all the isolates, a set of sequencing primers were designed spanning a region of the HA informative with regards to clade, strain, receptor binding motif, cleavability and glycosylation (Figure 1) and which specifically hybridize to H5N1. Sequences for relevant influenza viruses with known properties and virulence status were compiled for each sample from pyrograms. These sequences were then compared to known H5N1 sequences by alignment and visual inspection.

### Pyroseqencing

Biotinylated PCR product (5 ng/µl) from RT step from viral RNA (Figure 1) was immobilized onto 2.5 µl streptavidin-coated High Performance Sepharose beads (Amersham Biosciences, Piscataway, NJ) by incubation at room temperature for at least 10 minutes with agitation at 1400 rpm. Single-stranded DNA was obtained by washing the immobilized PCR product with 70% EtOH, denatured with 0.2 M NaOH, and washed with TA-Buffer (0.1 M Tris-Acetate, pH 7.6) using a Vacuum Prep Tool and Vacuum Prep Worktable (Biotage, Uppsala, Sweden). The beads were then suspended in 12 µl annealing buffer (10 mM Tris-acetate pH 7.75, 5 mM Mg-acetate) containing 0.3 pmol sequencing primer. Single-stranded DNA was hybridized to the sequencing primer by incubation at 90°C for 2 minutes, at 60°C for 5 minutes and at room temperature for 5 minutes.

Primed single-stranded PCR products were sequenced using PSQ™ HS96A System (Biotage). Sequencing was performed in a total volume of 12 µl using the PSQ 96 Gold kit (Biotage). Consensus pre-programmed dispensation orders were determined by integrating sequence information for each strain at a given sequencing site [34]. See figure 5 for a comparison of *de novo* and pre-programmed sequencing strategies. As shown, approximately 10 more nucleotides of sequence information are obtained with the pre-programmed method, because nucleotide incorporations are anticipated. This reduces the amount of time required as well as the accumulation of sequencing byproducts. Negative-control nucleotide dispensations were also included in the program to check for insertions and to measure background signals. The identity and number of nucleotide extension events were determined by automated measurement of the amount of light generated after incorporation of each dNTP.

Raw data were interpreted using software developed specifically for this purpose,“Classifier.” Classification of samples by strain is straightforward using the short sequence segments obtained. We use a Support Vector Machine [35] approach implemented in the statistical programming language R [36] to classify a given sample with statistical accuracy. This provides an automated sample identification tool, designed for eventual use with large numbers of source sequences [37], [38].

### Supplementary information

See http://bioel.stanford.edu/avianflu/index.html for detailed pyrosequencing results for all nine strains.
